# On PTV definition for glioblastoma based on fiber tracking of diffusion tensor imaging data

**DOI:** 10.1371/journal.pone.0227146

**Published:** 2020-01-06

**Authors:** Barbara Witulla, Nicole Goerig, Florian Putz, Benjamin Frey, Tobias Engelhorn, Arnd Dörfler, Michael Uder, Rainer Fietkau, Christoph Bert, Frederik Bernd Laun

**Affiliations:** 1 Department of Radiation Oncology, Universitätsklinikum Erlangen, Friedrich-Alexander-Universität Erlangen-Nürnberg, Erlangen, Germany; 2 Department of Neuroradiology, Universitätsklinikum Erlangen, Friedrich-Alexander-Universität Erlangen-Nürnberg, Erlangen, Germany; 3 Institute of Radiology, Universitätsklinikum Erlangen, Friedrich-Alexander-Universität Erlangen-Nürnberg, Erlangen, Germany; George Washington University, UNITED STATES

## Abstract

Radiotherapy (RT) is commonly applied for the treatment of glioblastoma multiforme (GBM). Following the planning target volume (PTV) definition procedure standardized in guidelines, a 20% risk of missing non-local recurrences is present. Purpose of this study was to evaluate whether diffusion tensor imaging (DTI)-based fiber tracking may be beneficial for PTV definition taking into account the prediction of distant recurrences. 56 GBM patients were examined with magnetic resonance imaging (MRI) including DTI performed before RT after resection of the primary tumor. Follow-up MRIs were acquired in three month intervals. For the seven patients with a distant recurrence, fiber tracking was performed with three algorithms and it was evaluated whether connections existed from the primary tumor region to the distant recurrence. It depended strongly on the used tracking algorithm and the used tracking parameters whether a connection was observed. Most of the connections were weak and thus not usable for PTV definition. Only in one of the seven patients with a recurring tumor, a clear connection was present. It seems unlikely that DTI-based fiber tracking can be beneficial for predicting distant recurrences in the planning of PTVs for glioblastoma multiforme.

## Introduction

A good outcome in radiation therapy requires a good definition of the planning target volume by means of imaging techniques like computer tomography (CT) or magnetic resonance imaging (MRI). For brain tumors, the use of MRI is advisable because of the good soft tissue contrast [1]. In case of glioblastoma (GBM, annual incidence of approximately 5000 cases in Germany, which has a population of roughly 83 million people [2]), MRI images are acquired before and after surgical removal of the visible tumor area for defining the clinical target volume (CTV). The “American Society of Clinical Oncology” recommends resection with consequent external beam radiation therapy (RT) [3, 4]. The target volume should include the area of the tumor before resection and the cavity after resection. Moreover, anatomical limits like edema, bones etc. should be taken into account when defining the target volume [3, 4]. The five year survival rate is between 25% and less than one percent depending on age and therapy [5, 6]. This stresses the need for better therapeutic approaches including, for example, a better target volume definition [7].

In about 20% of the glioblastoma patients, their recurrence was diagnosed as distant [8] and invasive glioma cells are found to migrate along myelinated fiber tracts of white matter [9]. The orientation of these tracts can be revealed with magnetic resonance diffusion tensor imaging (DTI) [10]. In DTI, the average water diffusion properties in an image voxel are detected and the larger diffusion parallel to white matter fiber tracts indicates their direction. Based on these data, white matter tracts can be reconstructed with fiber tracking techniques [11]. In a pioneering work, Krishnan et al. found that DTI-based fiber tracts connected primary glioma tumors with secondary lesions in eleven out of fourteen patients. They stated that this approach may be used to modify stereotactic radiotherapy target volumes to provide elongated treatment margins along the paths of elevated water diffusion, thereby creating a biologically better treatment plan that may reduce the incidence of progression [12].

Following this idea, we hypothesized that fiber tracking might be helpful for the definition of PTVs for radiation therapy of GBMs, and that the advances in scanner hardware and fiber tracking algorithms might lead to improved results. The aim of this work was to test this hypothesis.

## Material and method

### Patients

One hundred fifty-two patients were treated for brain tumors at the Department of Radiation Oncology at the University Hospital Erlangen from January 2015 until March 2017. Fifty-six patients were diagnosed with GBM, 30 of which developed a recurrence. Seven out of these 30 patients had a distant recurrence. These seven patients were included in this retrospective study.

The department of Neuroradiology and the institute of Radiology, which were involved in the patients´ treatment, agreed on using routine clinical data. All patients provided written informed consent when enrolled to the IRB approved study (No. 265_14B, Clinicaltrial ID: NCT02600065, ethics committee of the Friedrich-Alexander University Erlangen-Nürnberg).

### CT imaging

The planning CT for initial tumors as well as for recurrences was acquired at Sensation Open (Siemens Healthineers, Erlangen, Germany) with acquisition matrix 512 × 512. The voxel size depended on the reconstruction type. The slice thickness varied between 1 mm and 3mm and the pixel spacing varied between 0.7 × 0.7 mm^2^ and 0.98 × 0.98 mm^2^.

### MRI

The MRI scanning protocol included T1-weighted imaging (field of view (FoV) 192 mm × 320 mm, acquisition matrix 320 × 320, voxel size 0.72 × 0.72 × 5 mm^3^, repetition time (TR) 407 ms, and echo time (TE) 8.8 ms, 25 slices and 1 mm slice gap), T2 fluid attenuation inversion recovery imaging (FoV 230 mm^2^, matrix 512 × 512, voxel size 0.45 × 0.45 × 5 mm^3^, TR 9000 ms, TE 110 ms, and inversion recovery time 2500 ms, 25 slices and 1 slice mm gap), and a T2-MP-Rage sequence (FoV 256 × 256 mm^2^, matrix 256 × 256, voxel size 0.98 × 0.98 × 1 mm^3^, TR 1900 ms, TE 3.02 ms, inversion recovery time 1100 ms, 176 slices and 0.2 mm gap). The parameters of the echo planar DTI sequence were: b = 0 and 1000 s/mm^2^, 20 diffusion directions, FoV 256 × 256 mm^2^, matrix 128 × 128, voxel size 2 × 2 × 2 mm³, TR 10,500 ms, TE 93 ms, and an acquisition bandwidth of 1628 Hz/pixel. Data were acquired at a 1.5 Tesla MAGNETOM Aera (Siemens Healthineers, Erlangen, Germany) with a 20 channel head coil. MRI exams were conducted according to the standard clinical protocol: post resection, before RT and in three month intervals after the end of RT. For one patient, the first DTI was acquired after resection and during RT.

### Image registration

The radiation therapy treatment was planned with the treatment planning system (TPS) Pinnacle^3^ (Philips Healthcare, Eindhoven, The Netherlands), with the target volume (gross tumor volume according to ICRU Reports 50 and 62) definition performed in iPlan RT Image 4.1.2 (Brainlab, Munich, Germany) and based on rigidly registered CT and MRI acquired before RT (CT_plan_ and MRI_plan_) by experienced oncologists in the routine workflow. The initial gross tumor volume (GTV), PTV, edema, and organ at risk (OAR) (ROI_plan_) structures, as well as the CT_plan_, were exported to the research platform.

Before starting fiber tracking, the DTI data after surgical removal of the tumor (DTI_plan_) and the MP-rage of the recurrence (MRI_recurrence_) were registered rigidly on CT_plan_ by a radiation oncologist in iPlan RT Image 4.1.2 (Brainlab, Munich, Germany). The recurrence was also defined by a physician on CT_plan_ based on MRI_recurrence_. The recurrence structure (ROI_recurrence_) was exported. Then, CT_plan_ was rigidly registered to DTI_plan_ using the open source software Plastimatch (www.plastimatch.org) to get the transformation matrix (M_t_). M_t_ was applied on the ROI_plan_. As ROI_recurrence_ was defined on CT_plan_ for initial tumor treatment, it was also propagated to DTI_plan_ by M_t_.

### DTI processing, DTI analysis and fiber tracking

#### iPlan

Streamline tractography was conducted using the fiber tracking module of iPlan Image 4.1.2 (Brainlab AG, Munich, Germany). The DTI images were imported into iPlan and corrected for eddy currents [13]. iPlan uses the tensor deflection (TEND) fiber tracking algorithm with fractional anisotropy (FA) and value of minimum fiber length as stopping criteria [13]. We set the criteria to FA = 0.3 (recommendation made in a personal communication by Brainlab staff) and a minimum length of 50 mm. Three different seed regions were used: (i) GTV enlarged by 2 mm (seed GTV_+2mm_), (ii) GTV enlarged by 4 mm (seed GTV_+4mm_), (iii) recurrence volume (seed b). If more than one recurrence volume was present, the recurrence volumes were investigated separately. It was evaluated whether a connection between extended GTV volume and recurrence volume existed. If one or more of the tracked fibers entered both volumes, a connection was assumed to be present. Additionally, the strength of the connection was rated subjectively to be weak, middle, or strong.

#### MITK

Treatment planning CT (CT_plan_), initial GTV, recurrence structure (ROI_recurrence_) and the DTI (DTI_plan_) were co-registered and imported into the open source toolkit MITK-Diffusion (www.mitk.org). A discrete Gaussian noise filter with variance of 2 arbitrary units (a.u) was applied to the DTI raw data. Afterwards, a tensor map was generated via the module “Tensor” with a threshold of 50 a.u. on the b0 image to eliminate regions outside of the brain.

MITK Diffusion provides the opportunity of Gibbs tracking [14] and streamline tracking [11, 15–19]. The fibers were generated by streamline tracking with a FA threshold of 0.3 and the fiber assignment by continuous tracking (FACT) algorithm (MITK parameters f = 1 and g = 0, see also [17]) with minimal tract length of 50 mm. The same seed volumes as in iPlan were used.

In contrast to streamline tracking, Gibbs tracking is a global method for generating all fibers in the brain. Fibers are found by minimizing a cost function that guarantees consistency with the measured data and smoothness of fibers. This approach allows fibers to traverse fiber crossing regions. In contrast, using streamline techniques often results in premature fiber terminations in crossing regions because these regions are associated with small FA values in DTI. Gibbs tracking is able to overcome this limitation and can thus potentially unveil connections that are not found with streamline tracking.

Gibbs tracking in MITK features several parameters which can be set individually. The parameter particle length, particle width, particle weight and random seed were set to “auto”. Start Temperature was 0.1 a.u. and End Temperature was 0.001 a.u. The parameter balance of In/Ex Energy was set to 0. The parameter curvature threshold was 45° and the minimum fiber length was 50 mm. 10^8^ iterations were calculated.

After generating fibers by streamline and Gibbs tracking, they were extracted with help of the MITK “Fiber extraction” tool to find the fibers that pass the volumes of interest. I.e., the fibers generated by streamline tracking with seed GTV + enlargement were extracted for passing the recurrence volume. The fibers generated by seed b were extracted for passing GTV_+2mm_ or GTV_+4mm_. For Gibbs tracking, those fibers were extracted that passed GTV + enlargement and recurrence volume. The strength of the connection was rated subjectively in consensus by BW and FBL to be weak, middle, or strong. The rating “weak” was given if only a few connecting fibers were present, which subjectively could easily be attributed to origin from tracking errors. The rating “strong” was given if a doubtless connection was present. The rating “middle” was given otherwise.

## Results

**Fig 1:**
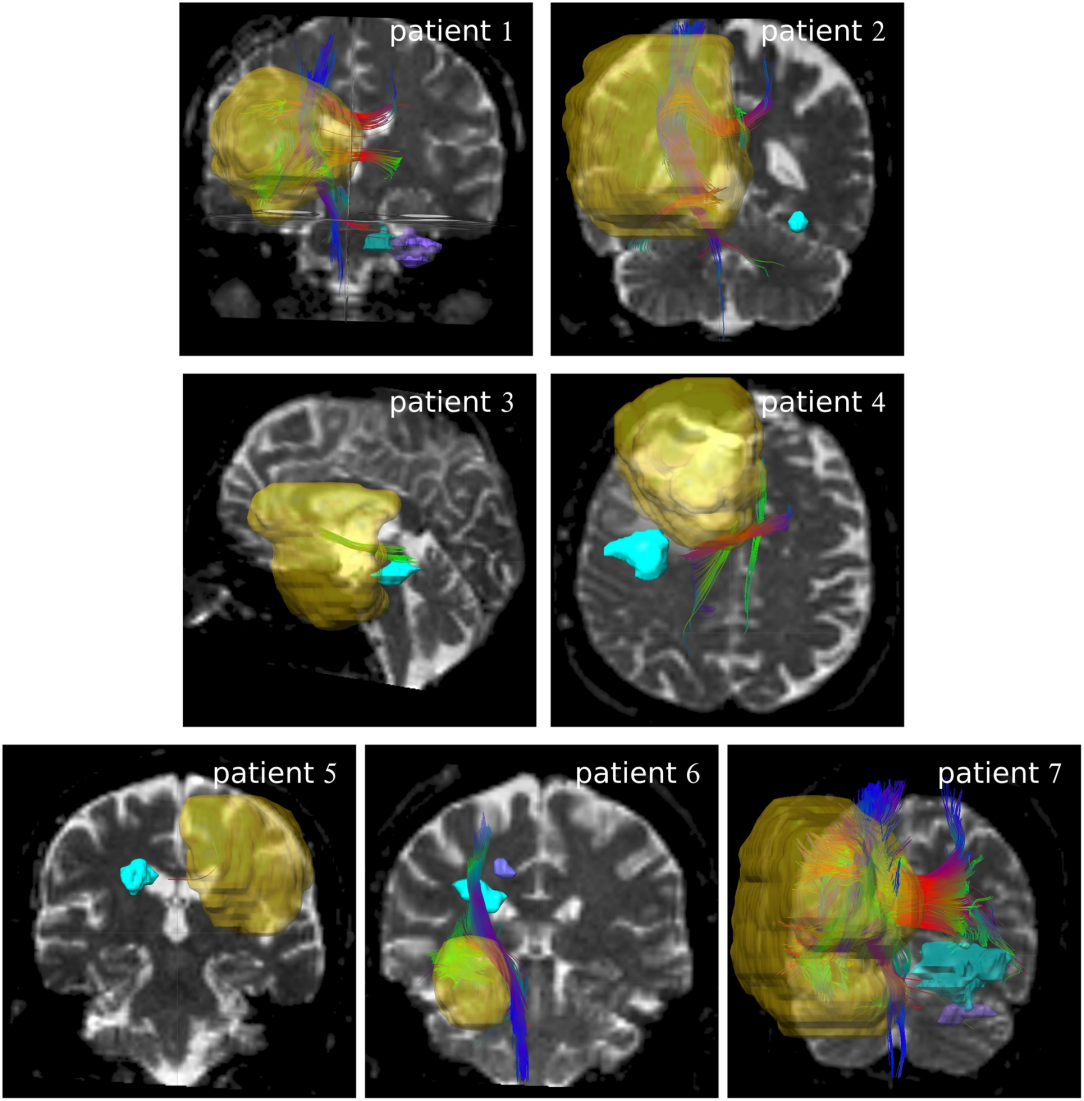
Fibers generated by iPlan. Fibers generated with GTV_+4mm_ as seed in iPlan. The yellow volume is the GTV_+4mm_. The recurrence volumes are displayed in cyan, and in purple if a second recurrence volume is presented. The fiber color indicates the fiber direction (blue: cranio-caudal, red: left-right, green: anterior-posterior).

Fig 1 shows the fiber tracking results obtained with iPlan with seed GTV_+4mm_. For patient 6, a strong connection between seed volume and recurrence volume 1 (displayed in cyan color) is visible. However, in all other cases, either only a weak connection is present, where the fibers are only touching the rim of the recurrence region (e.g. for patient 3) or no connection is present (e.g. for patient 2).

**Fig 2:**
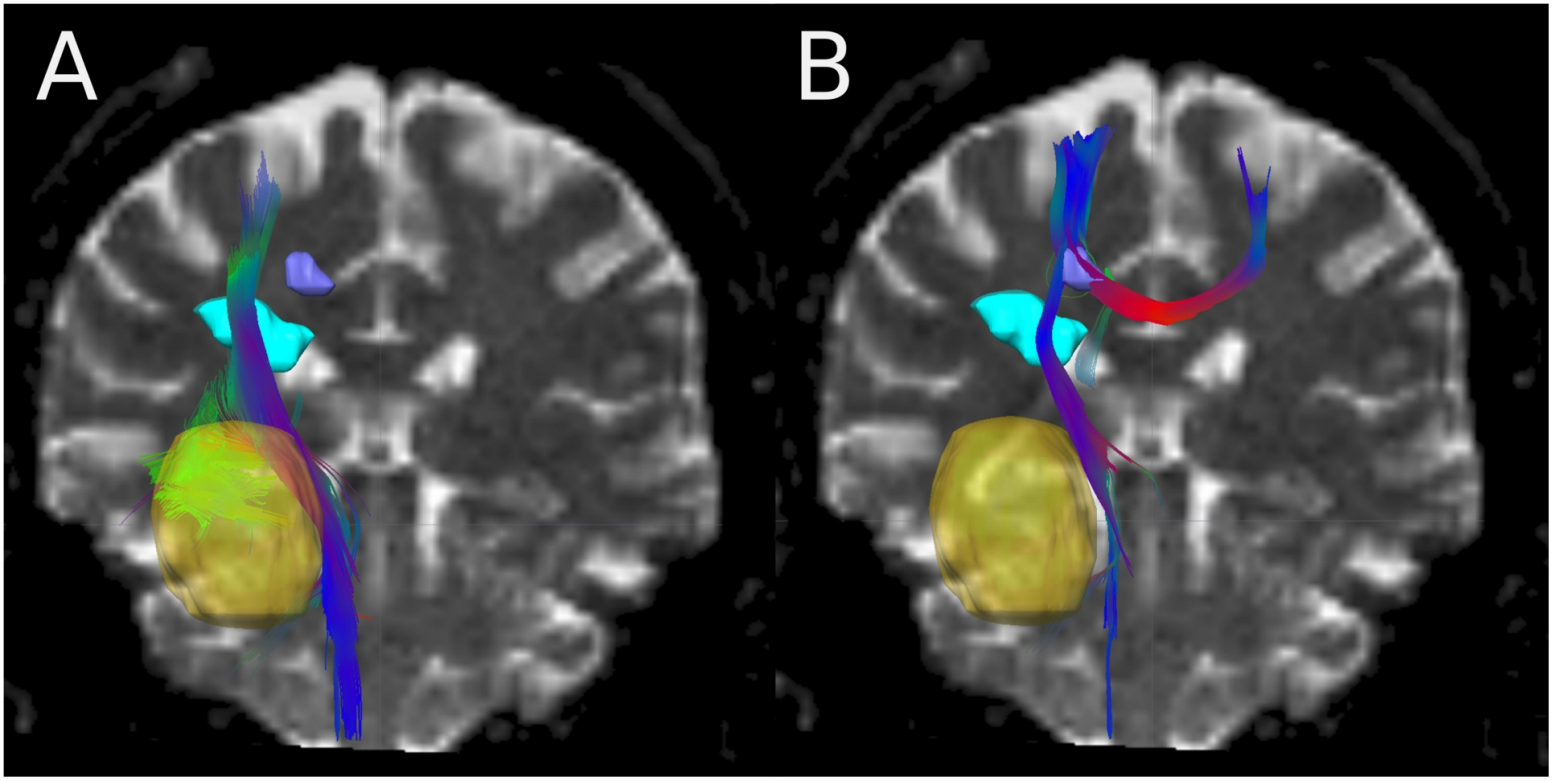
Fibers genareted by iPlan for patient 6. Result of streamline tracking in iPlan for patient 6 with different seeds: A) seed GTV_+4mm_ (yellow volume), B) seed recurrence 2 (purple volume) (B).

Fig 2 shows the fiber tracking results obtained with iPlan with different seed volumes for patient 6. Concerning the volume of the second recurrence displayed in purple, no connection is visible for seed GTV_+4mm_ (Fig 2a). However, a connection is present for seed b (purple volume, Fig 2b); i.e. it matters whether the tractography is started in the GTV or in the recurrence volume.

**Fig 3:**
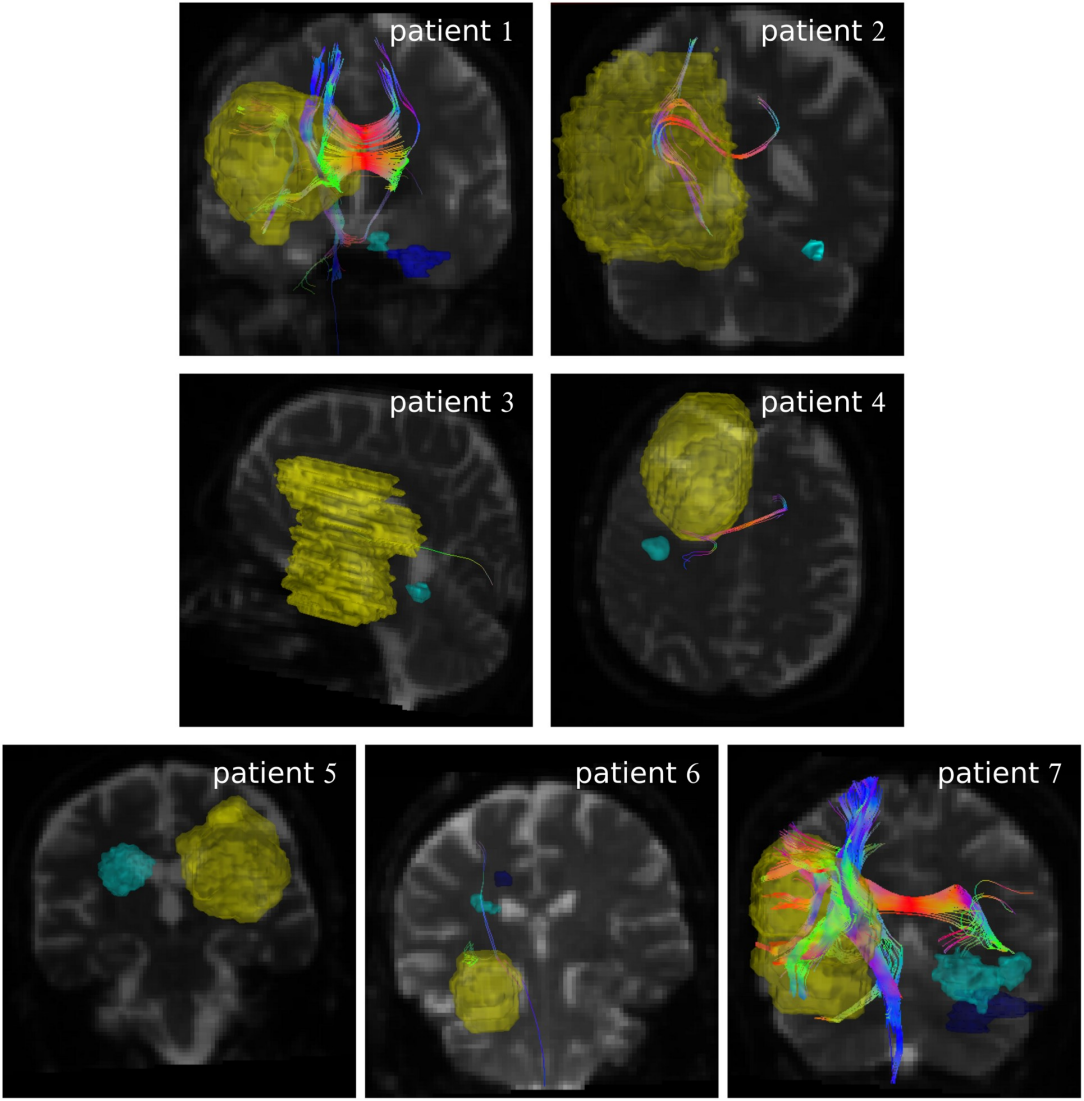
Fibers generated by streamline algorithm in MITK. Fibers generated with GTV_+4mm_ as seed by streamline algorithm in MITK. The yellow volume is the GTV increased by 4 mm. Recurrences are displayed in cyan and purple. Coloring of the fibers is identical to Fig 1.

Fig 3 is structured similar as Fig 1, but shows the tracking results of the MITK streamline tractography. Generally, less fibers were tracked than in iPlan although similar termination criteria were used. Consequently, even the strong connection observed for patient 6 in Fig 1 reduces to a weak connection. The different volume sizes in in Figs 1 and 3 arise from the registration onto the DTI dataset.

**Fig 4:**
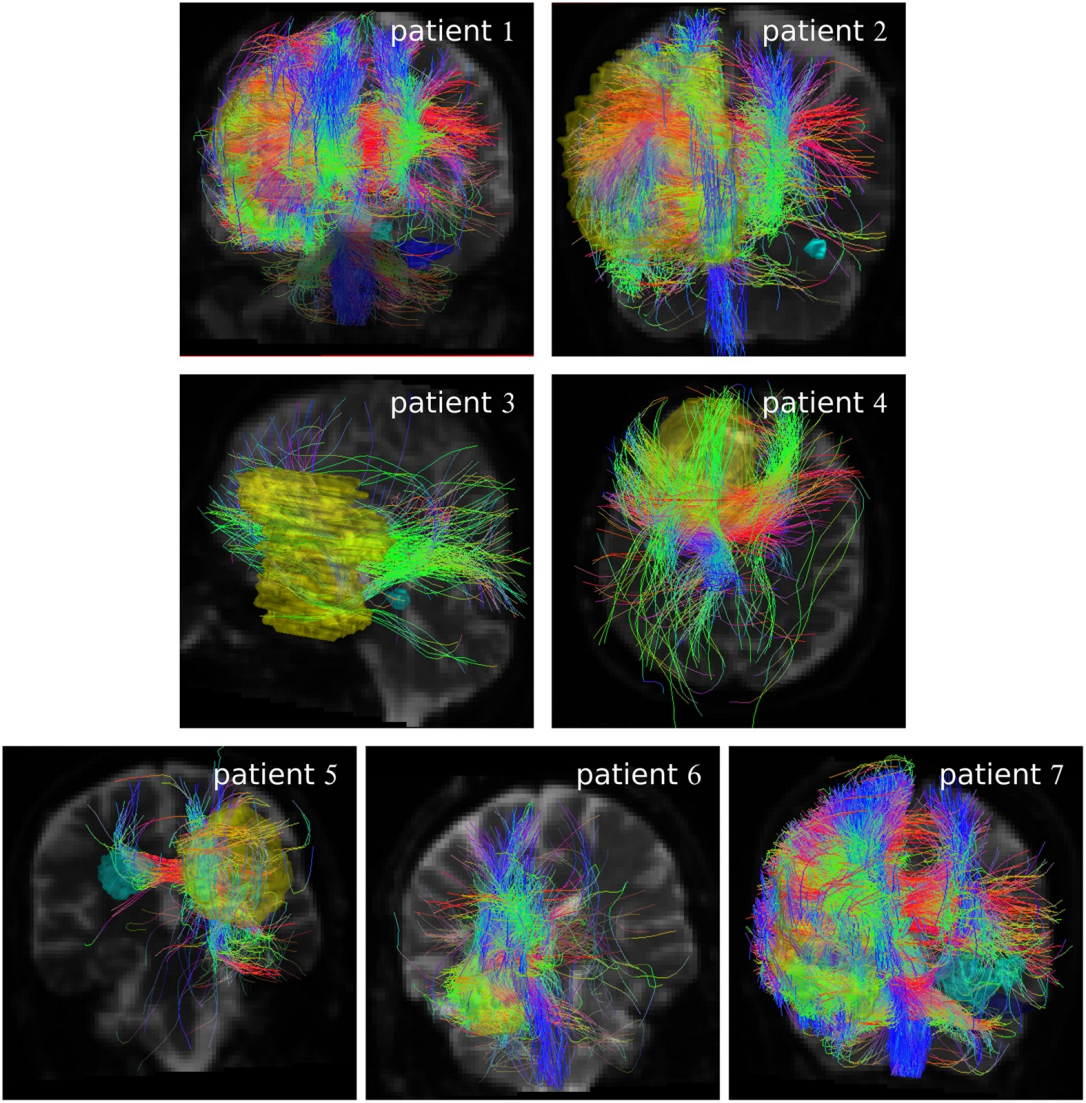
Fibers generated by Gibbs tracking in MITK. Result of Gibbs tracking for all patients after extraction. The fibers which pass GTV_+4mm_ (yellow volume) are shown. Recurrences are displayed in cyan and purple.

Fig 4 shows the result of the Gibbs tracking, showing all fibers that enter or pass the GTV_+4mm_. In comparison to the other two tracking approaches, much more fibers are generated often penetrating most of the brain volume. Consequently most recurrences feature some connection to the GTV_+4mm_.

**Fig 5:**
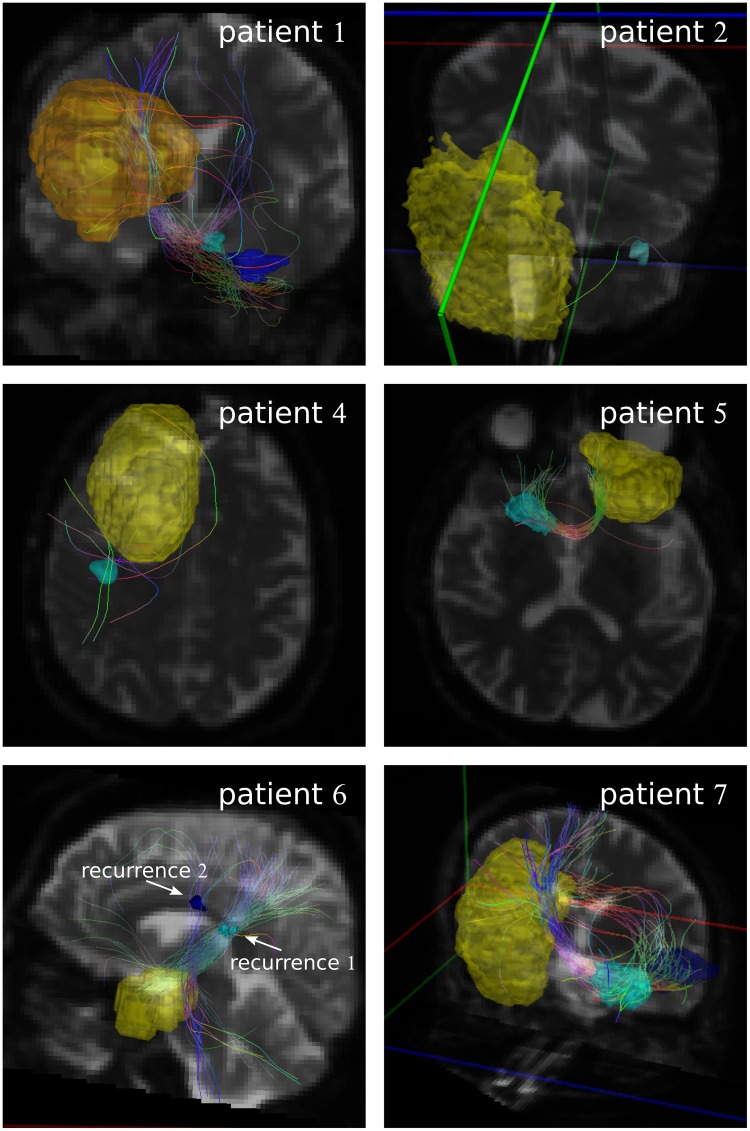
Connecting fibers generated by Gibbs algorithm. Connecting fibers from GTV_+4mm_ (yellow volume) to recurrences (cyan and purple volumes) generated by the Gibbs algorithm.

Fig 5 also shows Gibbs tracking results. Unlike in Fig 4, only those fibers are shown that enter or pass the GTV_+4mm_ and the recurrence volume. Thus, much less fibers are shown than in Fig 4. Still, a strong connection is again visible for patient 6. However, unlike in Figs 1 and 3, patients 1, 5, and 7 also have strong connections between GTV_+4mm_ and recurrence volumes.

Table 1 shows the quantitative and subjective evaluation of strength of the connection between GTV_+2mm_ (upper part) and GTV_+4mm_ (lower part), and the recurrences. For all recurrences, existence and strength of a detected connection strongly depended on the used algorithm and target volumes.

**Table 1:** Observed connection between extended GTV and recurrence volume.

**patient identification**	**iPlan**		**streamline algorithm in MITK**		**Gibbs algorithm in MITK**
	seed		seed		
	GTV_+2mm_	recurrence	GTV_+2mm_	recurrence	
1 (recurrence 1)	no	no	no	no	yes (weak, 7)
1 (recurrence 2)	no	no	no	no	yes (strong, 23)
2	no	no	no	no	yes (weak, 3)
3	no	yes (weak)	no	no	no
4	no	no	no	no	yes (weak, 1)
5	no	yes (strong)	no	no	yes (strong, 19)
6 (recurrence 1)	yes (strong)	yes (strong)	no	no	yes (strong, 55)
6 (recurrence 2)	no	no	no	no	yes (weak, 5)
7(recurrence 1)	no	no	no	no	yes (middle, 10)
7 (recurrence 2)	no	no	no	no	yes (strong, 43)
**patient identification**	**iPlan**		**streamline algorithm in MITK**		**Gibbs algorithm in MITK**
	seed		seed		
	GTV_+4mm_	recurrence	GTV_+4mm_	recurrence	
1 (recurrence 1)	no	no	no	no	yes (strong, 16)
1 (recurrence 2)	no	no	no	no	yes (strong, 41)
2	no	no	no	no	yes (weak, 1)
3	no	yes (middle)	no	no	no
4	no	no	no	no	yes (weak, 7)
5	no	yes (strong)	no	no	yes (strong, 34)
6 (recurrence 1)	yes (strong)	yes (strong)	yes (weak)	yes (middle)	yes (strong, 95)
6 (recurrence 2)	no	yes (weak)	no	no	yes (middle, 13)
7(recurrence 1)	no	no	no	no	yes (middle, 16)
7 (recurrence 2)	no	no	no	no	yes (strong, 48)

Observed connections between GTV extended by 2 mm / 4 mm and recurrence volume considering fibers generated by streamline algorithm in iPlan and MITK and by the Gibbs tracking algorithm in MITK with subjective rating of the strength of connection (weak, middle, strong). For the Gibbs tracking algorithm, the number of fibers passing GTV_+2mm_ / GTV_+4mm_ and recurrence is stated.

## Discussion

Of the 152 considered patients, seven had a distant recurrence. For one of these patients (patient 6), all used tracking approaches with seed GTV_+4mm_ found a strong connection between the GTV and one of the recurrences (recurrence 1). For the second recurrence of patient 6 and for the six other patients, the presence and strength of the connection depended strongly on the used tracking algorithm and on the seed volume definition.

The results indicate that the usefulness of fiber tracking methods for inclusion of distant recurrences in the definition of the PTV is rather limited for GBM patients. We used three different tracking approaches and observed the well-known strong dependency of tracking results on the used algorithm [20–23]. It may be argued that the variance of obtained results may be minimized by properly harmonizing the whole pipeline from acquisition protocol, seed volume definition, tracking method and tracking criteria, to the evaluation of the results. But the problem that the position of the recurrences was not predictable with our tracking data was present for each of the tested methods. For the two streamline tracking techniques, connections were mostly not present or only very weak. The Gibbs tracking approach yielded clear connection for more patients, which were, however, only identifiable retrospectively (Fig 5). At the time point of PTV delineation, only the GTV is known (as in Fig 4). However, the Gibbs tracking based on the GTV resulted in connections to almost all brain regions, which limits its predictive value. Thus, it seems unlikely that an optimization of parameters or the use of more advanced tracking algorithms–like machine learning techniques [15]–might help in this regard.

The reason for this negative finding is presumably twofold. First, it seems that a connection via large white matter fiber bundles is not a necessity for lesion spreading (as, for example, for patient 4). Second, the lesion may occur at quite distant position with respect to the primary tumor like in patient 2. Even if all distant lesions were connected to the primary tumor, knowing the fiber structure would not help for treatment planning because the lesions could still appear basically anywhere in the brain. This makes a well-conducted radiation dose boosting extremely challenging.

The results of Krishnan et al. were more promising than ours in terms of the predictive value of fiber tracking [12]. They included several glioma types with different therapies including only three patients post resection out of 14 patients in total, while we focused on GBMs after resection. This is, however, unlikely to explain the observed difference, since two out of three GBM patients had a strong connection in [12] for the distant secondary tumor group, which comes closest to our patient population. It seems more likely that the low number of patients in the two studies (14 in [12] and 7 in our study) and the associated large statistical uncertainty is the major reason for the observed differences.

DTI data have been used successfully in the context of glioma evaluation in several regards. For example, they have been used to adapt target volume definitions for GBM treatment planning [24–26], for modeling of brain tumor growth [27–30], to detect early malignant transformation of low-grade glioma [31], to differentiate between GBM and brain metastases [32], to use DTI-derived fiber tracking in surgery planning [33], and to detect the infiltration of the corpus callosum [34]. Our inability to properly connect most primary and secondary tumors does not diminish the value of DTI for these applications.

Potentially, other MRI techniques such as resting state functional MRI might represent potential venues for future research directions to relate the functional connectivity of the brain in GBM patients with the occurrence of new lesions [35].

One limitation of this study is the small patient number (seven patients with ten recurrences), which may be explained by the rareness of GBM and the low percentage of distant recurrences in GBM. Because of the surgical removal, a cavity occurred, which most likely resulted in a shift of brain anatomy [36–38]. Several physicians were involved in the definition of GTV and PTV treatment. If only one physician had planned GTV and PTV, the associated well-known inter-user variance could have been avoided [39]. One further limitation was the limited available scan time. Using 30 or more gradient directions would have stabilized the diffusion tensor calculation [40]. Advanced diffusion techniques that use higher b-values and generally require more scan time might also have improved the tractography results. These techniques include e.g. spherical deconvolution [41], neurite orientation dispersion diffusion imaging (NODDI) [42], higher order tensor reconstructions [43], or q-ball imaging [44]. The use of a higher field strength of 3 T or even 7 T would have been beneficial with respect to the signal to noise ratio and thus might have improved the tracking results [45].

## Conclusion

The study investigated the use of DTI based fiber tracking to predict the position of potential recurrences in GBM patients, which might be helpful for the definition of the PTV. The observed fibers were dependent on the used tracking algorithm and most of the recurrences could not be properly connected to the GTV in a prospective, i.e. predictive, manner. Based on the results of this study, it seems not recommendable to adapt the PTV definition for GBM patients including potential distance recurrences with fiber tracking based on the clinical DTI data acquisition techniques described in this study.
